# Green tea catechins and their metabolites in human skin before and after exposure to ultraviolet radiation^☆^^☆☆^^★^

**DOI:** 10.1016/j.jnutbio.2015.09.001

**Published:** 2016-01

**Authors:** Kayleigh A. Clarke, Tristan P. Dew, Rachel E.B. Watson, Mark D. Farrar, Joanne E. Osman, Anna Nicolaou, Lesley E. Rhodes, Gary Williamson

**Affiliations:** a School of Food Science and Nutrition, University of Leeds, Leeds, United Kingdom; b Photobiology Unit, Centre for Dermatology Research, Institute of Inflammation and Repair, University of Manchester, Manchester Academic Health Science Centre, Salford Royal NHS Foundation Hospital, Manchester, United Kingdom; c Manchester Pharmacy School, Faculty of Medical and Human Sciences, The University of Manchester, Manchester, United Kingdom

**Keywords:** AA, ascorbic acid, BMI, body mass index, C, catechin, *C*_max_, peak plasma concentration, EC, epicatechin, EG, ethyl gallate, EGC, epigallocatechin, ECG, epicatechin gallate, EGCG, epigallocatechin gallate, IS, internal standard, LC-MS/MS, liquid chromatography tandem mass spectrometry, LOQ, limit of quantification, Me, methyl, MED, minimal erythema dose, SE, standard error, *T*_max_, time to reach maximum plasma concentration, UVR, ultraviolet radiation, Green tea catechin conjugates, Polyphenols, Bioavailability, Ultraviolet radiation, Skin

## Abstract

Dietary flavonoids may protect against sunburn inflammation in skin. Preliminary reports using less complete analysis suggest that certain catechins and their metabolites are found in skin biopsies and blister fluid after consumption of green tea; however, it is not known if they are affected by solar-simulated ultraviolet radiation (UVR) or whether conjugated forms, with consequently altered bioactivity, are present. The present study tested the hypothesis that UVR affects the catechin levels in the skin of healthy volunteers after consumption of green tea and how catechins in the plasma are related to their presence in skin tissue samples. In an open oral intervention study, 11 subjects consumed green tea and vitamin C supplements daily for 3 months. Presupplementation and postsupplementation plasma samples, suction blister fluid and skin biopsies were collected; the latter two samples were collected both before and after UVR. A sensitive high-performance liquid chromatography/mass spectrometric assay was used to measure the intact catechin metabolites, conjugates and free forms. Seven green tea catechins and their corresponding metabolites were identified postsupplementation in skin biopsies, 20 in blister fluid and 26 in plasma, with 15 green tea catechin metabolites present in both blister fluid and plasma. The valerolactone, *O*-methyl-M4-*O*-sulfate, a gut microbiota metabolite of catechins, was significantly increased 1.6-fold by UVR in blister fluid samples. In conclusion, there were some common catechin metabolites in the plasma and blister fluid, and the concentration was always higher in plasma. The results suggest that green tea catechins and metabolites are bioavailable in skin and provide a novel link between catechin metabolites derived from the skin and gut microbiota.

## 1 Introduction

Daily consumption of green tea is associated with positive effects on human health [1], [2], [3], [4], [5]. Epidemiological studies have correlated green tea consumption with reduced cancer risk and cardiovascular disease [6], [7]. *In vitro* and *in vivo* studies also support the concept of protection against UVR-induced inflammation in skin by green tea consumption [8], [9], [10], [11], [12], [13], [14].

The skin is a metabolically active organ that acts as a barrier to protect the internal organs from the external environment, such as ultraviolet radiation (UVR) [15]. UVA and UVB penetrate the ozone layer, come into contact with the skin and initiate effects inside the cell, including changes in gene expression, generation of cytokines and proinflammatory lipid mediators and generation of reactive oxygen species [16], [17], [18], [19], [20]. Acute UVR exposure causes inflammation (sunburn) and photosensitivity, while repeated exposure leads to photoaging and carcinogenesis [21], [22]. Increased levels of sunlight exposure are a considerable health concern, with, for example, a significant increase in the malignant melanoma incidence observed between 1975 and 2010 in the United Kingdom [23]. The current use of topical sunscreen for UVR protection has limitations, including poor application and infrequent use outside of the holiday season [20]. Improving protection against UVR, possibly through a dietary source, may reduce the incidence of carcinogenesis.

Green tea contains catechins, a subgroup of the flavonoids, including catechin (C), epicatechin (EC), epigallocatechin (EGC), epicatechin gallate (ECG) and epigallocatechin gallate (EGCG). Previous bioavailability studies have identified that, following consumption, green tea catechins undergo phase II metabolism, and pharmacokinetic studies have shown the presence of the conjugated forms in plasma, with EGCG and ECG partially present as unconjugated forms [24], [25]. Recent bioavailability studies have focused mainly on the conjugated forms of catechin metabolites in urine and plasma samples [26], [27], [28]. The presence of green tea catechins in various tissues has also been identified *in vivo* [29], [30], including our previous pilot study [31]; however, the conjugated forms have not been assessed previously. This is important since the metabolites and conjugates of catechins have very different biological activities and transport properties compared to the parent compounds.

In our previous pilot study [31], we made a preliminary identification of 8 green tea catechins (presence or absence following enzyme deconjugation assays) in skin biopsy samples, after the daily consumption of green tea catechin supplements and vitamin C (at the equivalent of 2 cups of green tea) for 3 months. The present study utilized an improved method [32], which is applicable for monitoring both the free and conjugated forms of green tea catechins in biological samples, particularly for those containing endogenous β-glucuronidase and sulfatase activities, following daily green tea and vitamin C supplement consumption (at the equivalent of 5 cups of green tea). We used this method to evaluate their oral bioavailability in skin by obtaining and assessing skin biopsies and blister fluid (pre-UVR and post-UVR), plasma and urine to understand whether the presence of catechins in tissues can be related to plasma.

## 2 Materials and methods

### 2.1 Study design

A total of 12 subjects were recruited between September 2011 and January 2012 for the 3-month open oral intervention study conducted in the Photobiology Unit, Centre for Dermatology Research, Salford Royal NHS Foundation Hospital (Manchester, United Kingdom). Inclusion criteria were as follows: healthy, aged 18–65 years, sun-reactive skin type I/II and body mass index (BMI) <35 kg/m^2^. Exclusion criteria were as follows: history of skin cancer, photosensitivity disorder or atopy, sunbathing or use of sunbeds in the past 3 months, taking photoactive medication or nutritional supplements and drinking >2 cups of tea per day.

Subjects consumed supplements daily that were the equivalent of 5 cups of green tea. Green tea supplements were gelatine capsules each containing 450 mg green tea (180 mg green tea catechins) and a further set of capsules each containing 25 mg vitamin C. Subjects were administered 3 green tea and 2 vitamin C capsules twice daily (to consume with breakfast and the evening meal; total 1080 mg green tea catechins and 100 mg vitamin C daily) for 12 weeks. Vitamin C was added to stabilize the green tea extract in the gut lumen [33] and has no impact on UVR erythema [34]. During the course of the study, four sample types were collected; plasma, skin blister fluid, skin biopsies and urine (Table 1). Urine was collected over 24 h at four points during the intervention: baseline (day −1, presupplementation), day 0 (postsupplementation), week 6 and week 12. Plasma was collected for the pharmacokinetic analysis on the first day of supplementation (day 0) and again at 3 h postconsumption during week 12. All samples were stored at −80°C prior to analysis.

### 2.2 Ethics

Ethics approval was received from the North Manchester Research Ethics Committee (reference 08/H1006/79; clinicaltrials.gov identifier: NCT01032031). All subjects gave written informed consent and the study conformed to the Declaration of Helsinki principles.

### 2.3 UVR exposure and skin sampling procedures

Solar-simulated radiation (Newport Spectra Physics Ltd, Didcot, United Kingdom), mimicking the UVR emission of sunlight (290–400 nm; 95% UVA and 5% UVB), was used for all UVR exposures. The light source was located 10 cm away from the skin and a radiometer was used to calibrate the source and confirm consistency of application. To determine the sunburn threshold, skin was irradiated with a geometric series of 10 doses of UVR. After 24 h, the lowest dose that produced a visually discernible erythema was defined as the minimal erythema dose (MED). This determined the UVR dose that was subsequently used in the study; a dose that was 3 times the individual’s MED was applied to buttock skin 24 h prior to skin tissue and blister fluid sampling.

Skin was sampled at presupplementation baseline and 12 weeks postsupplementation, with UVR exposures performed on one buttock and the other buttock providing control unexposed samples. Suction blister and punch biopsy procedures were performed as described previously [18]. Skin biopsies (5 mm punch) were snap frozen in liquid nitrogen. Blister fluid was removed with a needle and combined with 25 μl NaH_2_PO_4_ [0.4 mol/L with 200 g/L ascorbic acid (AA) and 1 g/L EDTA, pH 3.6] then snap frozen in liquid nitrogen. All samples were stored at −80°C prior to analysis.

### 2.4 Pharmacokinetics

Pharmacokinetic analysis of green tea catechin uptake and metabolism was performed as follows (Table 1). Subjects were instructed to fast from midnight. The following morning, an 18-G cannula was inserted into the right antecubital fossa. A baseline blood sample was taken and then subjects consumed a standard breakfast consisting of flavonoid-free cereal (cornflakes) with semi-skimmed milk, one slice of white toast with butter and a glass of water. A single oral dose of green tea catechins (540 mg total green tea catechins) with 50 mg vitamin C was administered 5 min after consumption of breakfast. Further blood samples were taken at 0.5, 1, 1.5, 2, 3, 4 and 6 h postsupplement consumption with subjects consuming a standard lunch of a cheese and chicken sandwich on white bread and a slice of flavonoid-free cake following the 4-h sample. Plasma was separated by centrifugation, stored at −80°C and analyzed as described below.

### 2.5 Materials

EC, EGC, ECG, EGCG, C and taxifolin were purchased from Extrasynthése (Genay, France). Ethyl gallate (EG) and 3-methyl gallic acid were obtained from Apin Chemicals Ltd (Oxfordshire, United Kingdom), hippuric acid, benzoic acid and 3-hydroxybenzoic acid were purchased from Fluka (Dorset, United Kingdom); AA was from Sigma Aldrich (Dorset, United Kingdom) and syringic acid and gallic acid were purchased from Alfa Aesar (Lancashire, United Kingdom). All standards were high-performance liquid chromatography grade (>90%). EC-*O*-sulfate and EGC-*O*-glucuronide were synthesized using the method by Wong *et al.* [35]. A Millipore Q water purifying system (Millipore, Hertfordshire, United Kingdom) provided ultrapure water (≥18.2 MΩ cm at 25°C) for liquid chromatography tandem mass spectrometry (LC-MS/MS) analysis. Standard curves were generated for aglycones (free forms) by spiking standards into blank, presupplementation plasma and blister samples (*R*^2^>0.97). Recoveries were calculated by spiking known amounts of standards into blank, presupplement plasma, blister and biopsy samples (postcryopulverization) and relating the peak area from the spiked sample to the peak area from the direct analysis of an equivalent volume by LC-MS/MS.

### 2.6 Sample analysis

#### 2.6.1 Urine analysis for compliance

Urine was excreted into HCl-washed flasks containing AA (~1 g/L) and stored at −80°C prior to analysis. Compliance to the study was tested through monitoring the presence of EC-*O*-sulfate and EGC-*O*-glucuronide in the urine during the trial at the four time points and by counting residual supplements (Table 1). The urine samples were analyzed by LC-MS/MS according to our previous method [32].

#### 2.6.2 Plasma analysis

Plasma samples were removed from −80°C storage and thawed on ice. The whole procedure was performed on ice. The samples were gently vortexed before 380 μl (technical duplicates for each biological sample) was removed and placed in a 2 ml Eppendorf with 20 μl AA (22.5 mmol/L in water; final concentration of 1 mmol/L) and 50 μl EG (4 μg/ml in water). The samples were vortexed for 10 s and subsequently 1 ml of hexane (Sigma Aldrich, Dorset, United Kingdom) was added drop-wise to the Eppendorf tube. The samples were vortexed for 1 min and centrifuged at 17,000*g* (4°C) for 10 min. The top fat layer was removed to waste and the sample was placed back on ice; 500 μl of ice-cold ethyl acetate was added and the procedure continued as for urine. The only changes in relation to the urine protocol were the addition of 1.5 ml acetonitrile to the sample remaining after the ethyl acetate extraction (therefore the preweighed tube was a 2-ml Eppendorf) and the injection of 10 μl for LC-MS/MS analysis. Prepared samples were stored at −20°C and defrosted on ice before LC-MS/MS analysis.

#### 2.6.3 Blister fluid analysis

Blister samples were removed from −80°C storage and thawed on ice. The initial volumes were recorded (101±44 μl). The whole procedure was performed on ice and immediately analyzed by LC-MS/MS. Two technical replicates were performed for each biological sample when there was >80 μl initially. The samples were gently vortexed and briefly spun down. Blister fluid (40 μl) was added to an Eppendorf, along with 10 μl of 0.4 mol/L NaH_2_PO_4_ solution (containing 200 g/L AA, 1 g/L EDTA and 0.04 μg/10 μl EG). Ice-cold ethyl acetate (300 μl) was added and the sample followed the same procedure as for urine; however, 160 μl acetonitrile was used, the sample was only centrifuged at 17,000*g* (4°C) for 5 min and the samples were reconstituted back to 20 μl using water before vortexing for 1 min after reweighing the Eppendorf tubes. Of the sample, 18 μl was collected and added to the corresponding dried-down ethyl acetate tube with 2 μl taxifolin (20 μg/ml in 50% acetonitrile and 1% AA). The samples were sonicated for 5 min and 9.5 μl was added to two wells on a covered microwell plate and 5 μl of the sample was injected for LC-MS/MS analysis.

#### 2.6.4 Biopsy analysis

Skin biopsies were removed from −80°C storage and the initial weights were recorded using preweighed 2-ml Eppendorf tubes. The procedure was conducted on ice prior to immediate analysis by LC-MS/MS. The biopsies were held with sterilized tweezers over a waste beaker and flushed with ice-cold hexane to remove any blood and lipid present on the outer layer. After washing with hexane, a stainless-steel rod and base were placed into a vat of liquid nitrogen for approximately 1 min until effervescing ceased. This was removed and, using tweezers, a biopsy was placed into the vat of liquid nitrogen until effervescing stopped. The biopsy was removed, placed in between the rod and base and pulverized with a hammer (cryopulverization). The sample was immediately placed back into the 2-ml Eppendorf tube using a scalpel and tweezers and was stored at −80°C until required. In between each biopsy procedure, the scalpel and tweezers were resterilized using 70% ethanol and hot water.

Prior to LC-MS/MS analysis, each pulverized sample was further homogenized using a microhomogenizer (Ultra Turrax, IKA T10 Basic; IKA Ltd, Cheshire, United Kingdom). To the 2-ml Eppendorf tube containing the cryopulverized biopsy sample, 100 μl ice-cold chloroform (with 0.1 g/L butylated hydroxytoluene; Fisher Scientific Ltd, Leicestershire, United Kingdom) and 100 μl ice-cold sodium dithionite (0.3 mol/L, with 0.08 μg EG; Sigma Aldrich, Dorset, United Kingdom) were added. The biopsy was homogenized by shearing of the samples on dry ice, to reduce the heat produced by the sheering process. After homogenization (for approximately 5 min), the sample was maintained on dry ice for 2 min before centrifuging for 2 min at 17,000*g* (4°C). The top aqueous layer was removed to a new Eppendorf tube and 100 μl of 0.3 mol/L sodium dithionite (not containing EG) was added to the biopsy sample, which was then vortexed for 1 min. The sample was placed on dry ice for 2 min and centrifuged again with the resulting top aqueous layer being combined with the previous.

Ice-cold ethyl acetate (500 μl) was added and the sample followed the same procedure as for urine; however, 600 μl acetonitrile was used. The samples were reconstituted to 40 μl with water and 36 μl was added to the corresponding dried-down ethyl acetate Eppendorf, alongside 4 μl taxifolin (20 μg/ml in 50% acetonitrile and 1% AA). The samples were sonicated for 5 min and 18 μl was added to two separate wells on a covered microwell plate prior to LC-MS/MS analysis (injection volume: 10 μl).

#### 2.6.5 LC-MS/MS analysis

Reverse-phase LC-MS/MS was performed as previously described [32], using an Agilent 1200 SL system (Agilent Technologies, Dorset, United Kingdom) connected to a 6410 triple quadrupole LC-MS/MS with electrospray ionization on the negative mode, with multiple reaction monitoring (Agilent Technologies, Santa Clara, CA, USA) with a Kinetex C18 column (2.6 μm, 150×2.1 mm; Phenomenex, Cheshire, United Kingdom). As green tea catechin conjugates (sulfate and glucuronide) are not commercially available, catechin conjugates were analyzed using peak area, and free forms were analyzed relative to known standards, relative to the internal standard EG (2 μg/ml). A total of 55 metabolites (green tea catechins, metabolites and conjugated forms) were investigated, and the MS transitions are as previously reported [32].

### 2.7 Statistical analysis

Differences in GTC and metabolite concentrations presupplementation and postsupplementation from UVR-exposed and UVR-unexposed skin were compared using the Friedman test with *post hoc* Wilcoxon signed-rank tests or analysis of variance. *P*<.05 was considered to indicate a statistically significant difference. Data are presented as mean±standard error (SE) for *n*=11 subjects.

## 3 Results

### 3.1 Subjects

One subject was excluded from analysis as the subject could not provide blood samples. Thus, 11 subjects (six males, five females; median age, 21 years; range, 18–58 years) completed the study and no side effects were reported. BMI was median (range) 24.4 (18.2–33.8) at baseline, 24.9 (18.2–34.5) at 6 weeks and 24.3 (17.8–34.8) at 12 weeks, with no significant change.

### 3.2 Recovery of the metabolites and conjugates from skin and plasma samples

EC, EGC, EC-*O*-sulfate and EGC-*O*-glucuronide recoveries and the limit of quantification (LOQ) for the different sample types are shown in Table 2, Table 3. The coefficient of variance for the recovery of spiked catechins and conjugates from plasma, blister and skin biopsy samples ranged from 0.01 to 0.1, 0.03 to 0.2 and 0.1 to 0.2, respectively, indicating that sample processing resulted in suitable reproducibility. The majority of the standards were recovered between 90% and 100% in the biological samples, except for EGC-*O*-glucuronide in the blister and biopsy samples. The exact chemical substitution positions of the conjugate moieties (methyl ester, sulfate ester and glucuronic acid) of the green tea catechin metabolites are unknown in the results presented and will therefore be referred to as *O*-methyl-catechin-*O*-glucuronide or *O*-methyl-catechin-*O*-sulfate.

### 3.3 Plasma pharmacokinetics

**Fig. 1:**
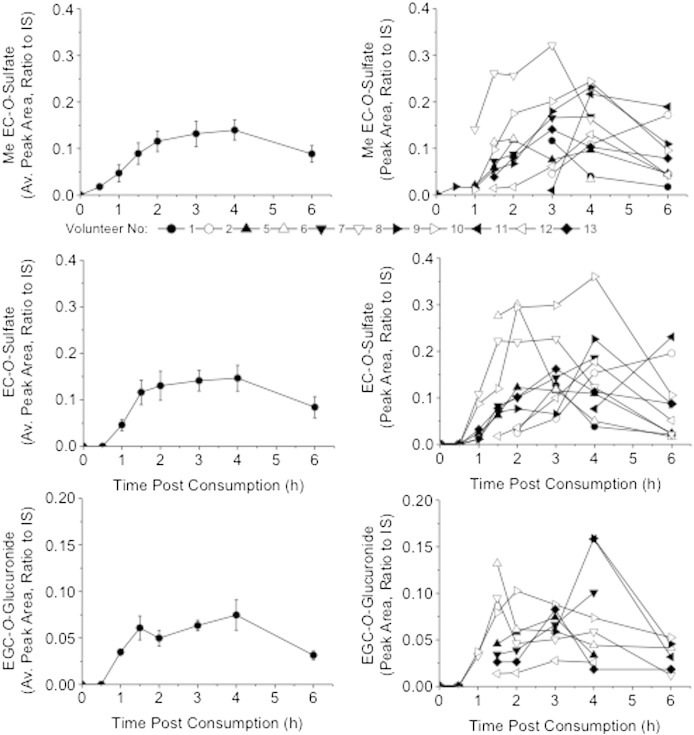
Average and individual pharmacokinetics for green tea catechin metabolites in the plasma of each volunteer postconsumption of green tea supplementation on day 0 (the first day of supplementation). The pharmacokinetics are for *O*-Me-*O*-EC-*O*-sulfate, EC-*O*-sulfate and EGC-*O*-glucuronide (assessed as peak area, relative to the internal standard, 2 μg/ml EG; mean±SE; *n*=11 subjects). EC, epicatechin; EG, ethyl gallate; EGC, epigallocatechin; IS, internal standard; Me, methyl.

The green tea catechin metabolites were detected after 1 h, except for EC-*O*-glucuronide, which was first detected 1.5 h postsupplement consumption. *T*_max_ values were calculated for the individual metabolites and ranged between 3 and 4 h. *C*_max_ values were calculated as peak area relative to the internal standard and ranged between 0.03 and 0.2. The pharmacokinetics for the 11 subjects were collated and presented as the mean±SE and also as individual pharmacokinetic curves. Example pharmacokinetics for *O*-methyl-*O*-EC-*O*-sulfate, EC-*O*-sulfate and EGC-*O*-glucuronide are displayed in Fig. 1.

There were 26 green tea catechin metabolites present in the plasma samples after 3 months supplementation, obtained 3 h postconsumption of the final dose of the supplement (Table 4). In comparison to the 3-h postconsumption sample on day 0 of supplementation, there were 7 additional metabolites present in the 3-month samples, including the gut microbiota metabolites, valerolactones (M4-*O*-sulfate, M4-*O*-glucuronide and M6/M6′-*O*-glucuronide) and a significant increase in EC-*O*-sulfate and M6/M6′-*O*-sulfate.

### 3.4 Blister fluid

We identified 20 green tea catechin metabolites in blister fluid, with few detected in baseline samples and the majority identified post green tea supplementation for 3 months (Table 5). The most prominent conjugated forms of EC and EGC, identified as the largest peak areas relative to the internal standard, were EC-*O*-sulfate, *O*-methyl-EC-*O*-sulfate and *O*-methyl-EGC-*O*-sulfate. For the valerolactones, M6/M6′-*O*-sulfate and *O*-methyl-M4-*O*-sulfate had the highest corresponding peak areas relative to the internal standard. *O*-Methyl-M4-*O*-sulfate was the only metabolite that was significantly increased in the postsupplementation UVR sample compared to the no-UVR sample.

### 3.5 Skin biopsy

Metabolites identified in the skin biopsy samples postsupplementation were *O*-methyl-EC-*O*-sulfate, gallic acid-*O*-glucuronide, *O*-methyl-gallic acid-*O*-sulfate, *O*-methyl-gallic acid-*O*-glucuronide, quercetin and M6 (Table 6). M6 was present in each sample from one volunteer and was the only metabolite identified in the presupplementation samples. No significant difference was found between the green tea catechin conjugates and metabolites present in the pre-UVR and post-UVR skin biopsies.

### 3.6 Bioavailability of green tea catechin metabolites

**Fig. 2:**
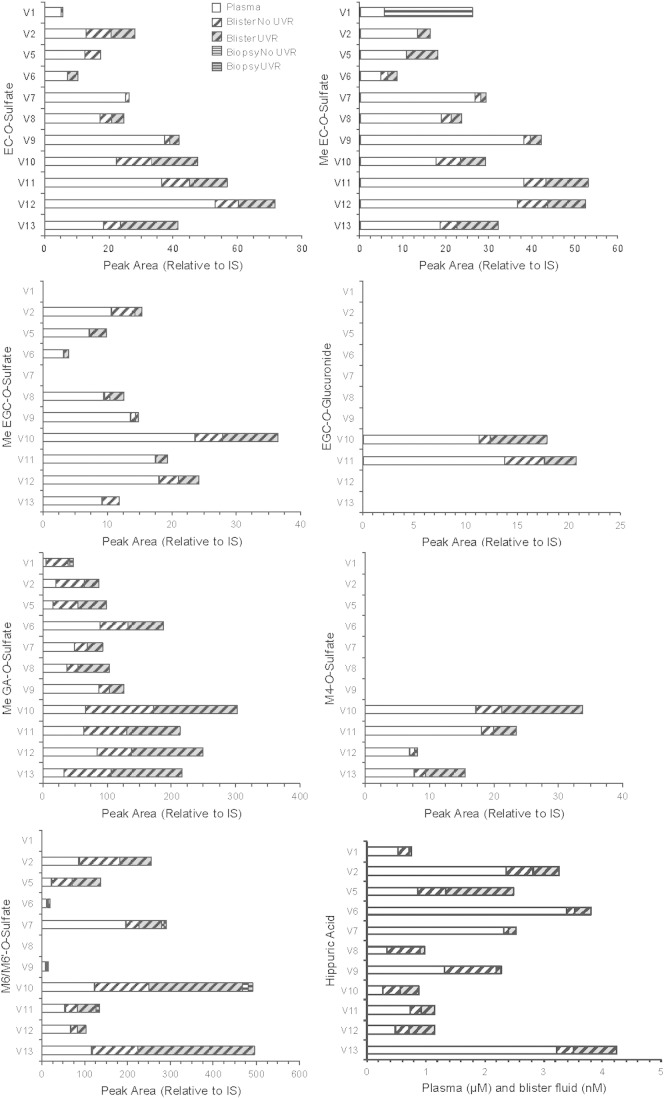
Identification of green tea catechin metabolites for the individual subjects in plasma, blister fluid and biopsy samples postsupplementation (after 3 months), with or without UVR. As peak areas for the conjugated green tea metabolites in the plasma were larger, plasma is presented as peak area ×10^−2^, blister analysis as peak area ×10^−6^ and biopsy analysis as peak area ×10^−7^ (relative to the internal standard, 2 μg/ml EG). Hippuric acid is presented as micromolars (μM) for plasma data and as nanomolars (nM) for blister analysis. The metabolites identified in subjects for more than one biological sample that are displayed are EC-*O*-sulfate, *O*-Me-EC-*O*-sulfate, *O*-Me-EGC-*O*-sulfate, EGC-*O*-glucuronide, *O*-Me-GA-*O*-sulfate, M4-*O*-sulfate, M6/M6′-*O*-sulfate and hippuric acid. EC-*O*-glucuronide, *O*-Me-M4-*O*-sulfate, M4-*O*-glucuronide, M6/M6′-*O*-glucuronide and benzoic acid-*O*-sulfate were also present in more than one biological sample. EGCG and 3-hydroxybenzoic acid sulfate were identified in more than one biological sample for one volunteer only. Only *O*-Me-EC-*O*-sulfate, *O*-Me-GA-*O*-sulfate and M6/M6′-*O*-sulfate graphs show biopsy data. EC, epicatechin; EG, ethyl gallate; EGC, epigallocatechin; EGCG, epigallocatechin gallate; GA, gallic acid; IS, internal standard; Me, methyl; V, volunteer number.

There were 15 green tea catechin metabolites (both free and conjugated forms) present in at least two of the biological samples, which were EC-*O*-sulfate, *O*-methyl-EC-*O*-sulfate, EGC-*O*-glucuronide, *O*-methyl-EGC-*O*-sulfate, EGCG, *O*-methyl-gallic acid-*O*-sulfate, M4-*O*-sulfate, M4-*O*-glucuronide, *O*-methyl-M4-*O*-sulfate, M6/M6′-*O*-sulfate, M6/M6′-*O*-glucuronide, benzoic acid-*O*-sulfate, 3-hydroxybenzoic acid-*O*-sulfate and hippuric acid (Fig. 2). The majority of the metabolites that were present in blister fluid were also present in the plasma. There were only two metabolites that were identified in all plasma, blister fluid and biopsy samples: *O*-methyl-gallic acid-*O*-sulfate (in six subjects) and M6/M6′-*O*-sulfate (in three subjects).

## 4 Discussion

The aim of the study was to determine whether green tea catechins and metabolites could be identified in human skin samples (blister fluid and biopsies), whether the bioavailability in skin could be related to their presence in plasma and whether the amounts were affected by UVR exposure. In total, there were 15 metabolites identified after 3 months supplementation in at least two of the three samples (plasma, blister fluid and biopsies) obtained 3 h post green tea supplement consumption, with only *O*-methyl-gallic acid-*O*-sulfate and M6/M6′-*O*-sulfate identified in all three samples. In accordance with other green tea bioavailability studies, there was a large interindividual variation [36], [37].

The blister fluid is a vacuum-pressure-induced collection of the extracellular fluid surrounding cells and may contain a contribution from plasma. As expected, the majority of the metabolites identified in more than one tissue sample were present within blister fluid and plasma, with limited metabolites identified in biopsy samples. It is possible that the metabolites did not enter the skin cells, that the metabolites present within the skin biopsy samples were bound to intracellular proteins or that they were further metabolized. A significant finding of the study was the increase in *O*-methyl-M4-*O*-sulfate observed in the postsupplementation UVR blister fluid sample compared to no UVR, and this difference may be an increase in response to the external stress. M4 is a hydroxyl-phenyl-valerolactone that derives from microbial metabolism of EGC [38] and, as suggested in our previous study, is a biomarker of green tea consumption [31]. M6 (derived from EC consumption) was identified in all biopsy samples (presupplementation and postsupplementation) for one volunteer; however, this was possibly due to dietary consumption of chocolate, black tea or wine and good dietary sources of EC, on the previous day. As a 24-h dietary recall was not undertaken, the other dietary sources of EC were not recorded.

After 3 months daily consumption, the metabolites quantified in the plasma were in line with other studies [25], [27], [28], [29]. The *C*_max_ of EGCG was 45 nmol/L when normalized to a 50 mg dose of EGCG in the green tea supplements, which is also in line with previous studies of plasma (reviewed by Williamson *et al.* [39]). For the first time, quercetin-*O*-sulfate, kaempferol-*O*-glucuronide, syringic acid-*O*-sulfate, 3-hydroxybenzoic acid-*O*-sulfate and benzoic acid-*O*-sulfate were identified in plasma samples post green tea consumption. The identification of quercetin and kaempferol after consumption of green tea has been assessed previously in plasma, but only as aglycones post enzyme deconjugation [40], [41].

Our preliminary study [31] identified 8 metabolites in the skin biopsy samples. Contrasting with the current work, the free-form (unconjugated) derivatives of the metabolites present following consumption of green tea supplements were only assessed after enzyme deconjugation, and the tissue examined was limited to the dermal layer of the skin. Only one catechin was identified in a biopsy for the current study, whereas EC, EGC, 4′-*O*-methyl-EGC and EGCG were tentatively identified in the pilot study. This is possibly due to the accumulation of free-form derivatives following enzyme deconjugation, which may result in a higher LC-MS/MS response in comparison to the lower concentrations of the individual conjugates for one particular catechin. However, the current methodology gives a clearer and more realistic overview of both the free-form and conjugated derivatives of the green tea catechin metabolites identified, even though some of the individual metabolites may consequently be under the limit of detection.

This is the first bioavailability study to identify the presence of both free and conjugated forms of green tea catechins and metabolites in skin tissue, blister fluid and plasma. The method reported good recoveries for the majority of the compounds assessed. The main limitation of the study is the lack of commercially available green tea catechin and metabolite conjugated standards, and therefore, the results obtained are speculative. Other analytical methods, such as NMR, could be used to improve the identification of the metabolites within the samples. Overestimation may occur as the conjugated forms ionize more efficiently and the precise response factors are also unknown; therefore, the green tea catechins were reported relative to standards and the conjugated forms were reported as peak areas relative to the internal standard to improve the quality of the data.

In summary, the results of the study suggest that green tea catechin metabolites can reach the skin postsupplementation in the unconjugated (free) form and as conjugated derivatives, as assessed using a dual combination method to identify both [32], with an increase in *O*-methyl-M4-*O*-sulfate following UVR exposure in blister fluid. As expected, the majority of metabolites were present in the conjugated form. There was a clear overlap between the metabolites present in the plasma samples collected 3 h postconsumption and the blister fluid. Therefore, any lowering of UVR-induced inflammatory markers or improvement to skin health reported in future studies following long-term green tea supplementation could potentially be associated with the metabolites reported in the present study.

## Figures and Tables

**Table 1:** Green tea catechin and vitamin C supplementation and sample collection protocol.

Time point	Protocol
Day −2	24 h urine collection (baseline)
Assess MED and apply 3× MED for UVR exposed samples
Day −1	Suction blister (1× no UVR, 1× 3× MED UVR)
Punch biopsy (1× no UVR, 1× 3× MED UVR)
Day 0 (supplementation period commenced)	24 h urine collection (postingestion)
Plasma pharmacokinetics
(preingestion: 0 h)
(postingestion: 0.5, 1, 1.5, 2, 4, 6 h)
24 h urine collection
Week 6	24 h urine collection
Week 12, day −1	Apply 3× MED UVR
Week 12	Suction blister (1× no UVR, 1× 3× MED UVR; initiated 1 h and samples obtained ~3 h postingestion)
Punch biopsy (1× no UVR, 1× 3× MED UVR; samples obtained 3 h postingestion)
Plasma collection (3 h postingestion)

**Table 2:** Recovery of green tea catechins and related metabolites in the plasma and skin samples.

	Recovery		
Compound	Plasma	Blister fluid	Skin biopsy
	%	%	%
EC	107±14	90±2	102±27
EGC	103±3	75±8	105±6
EC-*O*-sulfate	117±2	86±2	108±15
EGC-O-glucuronide	97±2	32±5	62±13
EG	93±1	104±13	96±4

Recovery data are expressed as percentages of corresponding standards (mean±SE). Recoveries were performed in technical triplicate at a minimum of three concentrations. EC, epicatechin; EG, ethyl gallate; EGC, epigallocatechin.

**Table 3:** LOQ for the free-form green tea catechin metabolites (related to sulfate or glucuronide forms) for each biological sample.

	LOQ		
Compound	Plasma	Blister fluid	Skin tissue biopsy
	pmol/L	pmol/L	pmol/mg tissue
EC	2	20	NA
3′-*O*-Me EC	1	20	NA
4′-*O*-Me EC	0.1	NA	1
EGC	0.2	10	NA
3′-*O*-Me EGC	1	NA	NA
4′-*O*-Me EGC	0.6	230	NA
ECG	0.1	NA	NA
EGCG	0.1	NA	NA
Gallic acid	40	110	NA
3-*O*-Me gallic acid	2	10	10
Quercetin	20	60	30
Kaempferol	110	NA	NA
M6	3	30	9
Syringic acid	20	40	NA
3-Hydroxybenzoic acid	5	30	NA
Benzoic acid	50	590	NA
Hippuric acid	0.4	40	NA

EC, epicatechin; ECG, epicatechin gallate; EGC, epigallocatechin; EGCG, epigallocatechin gallate; Me, methyl; NA, not assessed using free-form standards, as the free-form or associated sulfate or glucuronide form was not identified in a sample.

**Table 4:** Changes in the concentration of green tea catechin metabolites present in plasma 3 h postingestion of a green tea supplement on the first day (day 0) and after 3 months of supplementation.

Compound	Ingestion of green tea supplement	
3 h, day 0	3 h, 3 months
Conjugated form	Peak area ratio	Peak area ratio
EC-*O*-sulfate	14±2 (10)	23±4⁎ (11)
EC-*O*-glucuronide	4±1 (7)	5±1 (9)
*O*-Me-EC-*O*-sulfate	13±2 (10)	21±4⁎ (11)
EGC-*O*-sulfate	4±1 (3)	3.3±0.7 (2)
EGC-*O*-glucuronide	5.9±0.7 (9)	8±2 (10)
*O*-Me-EGC-*O*-sulfate	10±2 (9)	12±2 (11)
*O*-Me-EGC-*O*-glucuronide	1.8±0.5 (3)	1.9±0.4 (6)
ECG-*O*-sulfate	ND	3 (1)
Gallic acid-*O*-sulfate	ND	4±1 (2)
*O*-Me-gallic acid-*O*-sulfate	40±7 (11)	50±9 (11)
Quercetin-*O*-sulfate	ND	2.5±0.4 (3)
Kaempferol-*O*-glucuronide	ND	3 (1)
M4-*O*-sulfate	ND	10±2 (9)
M4-*O*-glucuronide	ND	2.0±0.7 (5)
*O*-Me-M4-*O*-sulfate	1 (1)	38±8 (9)
M6/M6′-*O*-sulfate	10±4 (8)	60±20⁎ (11)
M6/M6′-*O*-glucuronide	ND	5±2 (7)
*O*-Me-M6/M6′-*O*-sulfate	4±2 (3)	5.6±0.4 (2)
Benzoic acid-*O*-sulfate	19±6 (11)	14±3 (10)
3-Hydroxybenzoic acid-*O*-sulfate	1 (1)	6±3 (3)
Syringic acid-*O*-sulfate	7±6 (4)	3±1 (5)
Free form	μM	μM
EGC	0.2 (1)	0.24±0.01 (2)
ECG	0.08 (1)	0.25±0.04 (2)
EGCG	0.10±0.02 (4)	0.3±0.1 (4)
3-Hydroxybenzoic acid	4±1 (5)	9±3 (7)
Hippuric acid	1.4±0.4 (10)	1.6±0.2 (11)

Data for the conjugated forms are the average peak area ratio (×10^−2^) of the compound relative to the internal standard, EG (2 μg/ml). Data are presented as mean±SE, and the data in brackets are the number of subjects the metabolite was identified in. When no SE is presented, the metabolite was only detected in one volunteer. Samples were analyzed in biological duplicate and technical triplicate. EC, epicatechin; ECG, epicatechin gallate; EGC, epigallocatechin; EG, ethyl gallate; EGCG, epigallocatechin gallate; Me, methyl; ND, not detected.

⁎ *P*<.05 compared to day 0 (analysis of variance).

**Table 5:** Green tea catechin metabolites in blister fluid presupplementation and postsupplementation, with or without UVR exposure.

Compound	Amount/blister fluid collected			
Pre, No UVR	Pre, UVR	Post, No UVR	Post, UVR
Conjugated form	Peak area ratio/μl	Peak area ratio/μl	Peak area ratio/μl	Peak area ratio/μl
EC-*O*-sulfate	ND	ND	5±3 (10)	9±5 (8)
EC-*O*-glucuronide	ND	ND	ND	3±2 (3)
*O*-Me-EC-*O*-sulfate	ND	ND	4±2 (8)	5±3 (10)
EGC-*O*-glucuronide	ND	ND	2±2 (2)	3.3±0.2 (2)
*O*-Me-EGC-*O*-sulfate	ND	ND	3±2 (6)	2±2 (7)
*O*-Me-EGC-*O*-glucuronide	ND	ND	1 (1)	2 (1)
Gallic acid-*O*-glucuronide	1±1 (2)	12 (1)	ND	ND
*O*-Me-gallic acid-*O*-sulfate	57±37 (11)	90±73 (11)	46±28 (11)	55±38 (11)
M4-*O*-sulfate	ND	ND	2±1 (4)	5±3 (4)
M4-*O*-glucuronide	ND	ND	0.7±0.4 (2)	4 (1)
*O*-Me-M4-*O*-sulfate	ND	ND	9±6 (6)	14±8⁎ (7)
M6/M6′-*O*-sulfate	7±4 (3)	15±16 (2)	46±47 (10)	78±88 (9)
M6/M6′-*O*-glucuronide	ND	ND	3±3 (4)	6±5 (4)
Benzoic acid-*O*-sulfate	ND	ND	2 (1)	4±4 (3)
Syringic acid-*O*-sulfate	ND	2 (1)	ND	ND
Syringic acid-*O*-glucuronide	ND	2 (1)	ND	ND
3-Hydroxybenzoic acid-*O*-sulfate	ND	ND	8 (1)	8±6 (3)
Free form	pmol/μl	pmol/μl	pmol/μl	pmol/μl
EGCG	20 (1)	14 (1)	9 (1)	ND
Hippuric acid	340±380 (11)	670±800 (11)	230±150 (11)	350±330 (11)
Benzoic acid	10,550 (1)	30,570 (1)	ND	ND

Data are the average peak area ratio relative to the internal standard (EG; 2 μg/ml) ×10^−6^ in total blister fluid extracted for the conjugated forms. Samples were analyzed pre and post 12 weeks supplementation and with or without UVR irradiation. Samples were analyzed in biological duplicate and technical duplicate; mean±SE. The data in brackets are the number of subjects in which the metabolite was identified. EC, epicatechin; EGC, epigallocatechin; EG, ethyl gallate; EGCG, epigallocatechin gallate; ND, not detected.

⁎ *P*<.05 compared to post no UVR (two-tailed Wilcoxon signed-rank test).

**Table 6:** Green tea catechin metabolites in skin biopsies presupplementation and postsupplementation, with or without UVR exposure.

Compound	Amount/biopsy weight			
Pre, No UVR	Pre, UVR	Post, No UVR	Post, UVR
Conjugated form	Peak area ratio/mg	Peak area ratio/mg	Peak area ratio/mg	Peak area ratio/mg
*O*-Me-EC-*O*-sulfate	ND	ND	20 (1)	ND
Gallic acid-*O*-glucuronide	ND	ND	8 (1)	9 (1)
*O*-Me-gallic acid-*O*-sulfate	ND	ND	ND	6 (1)
*O*-Me-gallic acid-*O*-glucuronide	ND	ND	ND	14 (1)
M6/M6′-*O*-sulfate	ND	ND	9±5 (2)	8±2 (3)
Free form	pmol/mg	pmol/mg	pmol/mg	pmol/mg
Quercetin	ND	ND	120 (1)	ND
M6	240 (1)	370 (1)	230 (1)	80 (1)

Data are the average peak area ratio relative to internal standard (EG; 2 μg/ml) ×10^−7^, standardized to the initial weight of the biopsy. Samples were analyzed presupplementation and postsupplementation and with or without UVR irradiation. Samples were analyzed in technical duplicate; mean±SE; data in brackets are the number of subjects in which the metabolite was identified. EC, epicatechin; EG, ethyl gallate; Me, methyl; ND, not detected.
